# Right Frontotemporal Cortex Mediates the Relationship between Cognitive Insight and Subjective Quality of Life in Patients with Schizophrenia

**DOI:** 10.3389/fpsyt.2018.00016

**Published:** 2018-02-02

**Authors:** Shenghong Pu, Kazuyuki Nakagome, Masashi Itakura, Hiroaki Ohtachi, Masaaki Iwata, Izumi Nagata, Koichi Kaneko

**Affiliations:** ^1^Division of Neuropsychiatry, Department of Brain and Neuroscience, Faculty of Medicine, Tottori University, Yonago, Japan; ^2^National Center of Neurology and Psychiatry, Kodaira, Japan; ^3^National Hospital Organization, Tottori Medical Center, Tottori-shi, Japan

**Keywords:** cognitive insight, near-infrared spectroscopy, verbal fluency task, frontotemporal function, quality of life, schizophrenia

## Abstract

Although prior studies identified a relationship between cognitive insight and subjective quality of life (QOL) in patients with schizophrenia, the brain regions mediating this relationship remain unknown. Recent studies have shown that the ventrolateral prefrontal cortex may be particularly important for cognitive insight in individuals with schizophrenia. Here, we examined whether frontotemporal function mediates the relationship between cognitive insight and QOL in 64 participants, including 32 patients with schizophrenia and 32 healthy controls. Cognitive insight was measured using the Beck Cognitive Insight Scale (BCIS), while participants’ subjective QOL was assessed using the Medical Outcomes Study 36-item Short-form Health Survey. Frontotemporal function was evaluated during a verbal fluency task using multichannel near-infrared spectroscopy. Consistent with previous findings, we found that frontotemporal function was impaired in patients with schizophrenia. Interestingly, our data also revealed that the right ventrolateral PFC and the right anterior part of the temporal cortex significantly mediated the relationship between the self-reflectiveness (SR) subscale of the BCIS and subjective QOL. These findings suggest that cognitive insight, particularly SR, is associated with subjective QOL in patients with schizophrenia *via* right frontotemporal function. The findings of this study provide important insight into a QOL model of schizophrenia, which may guide the development of cost-effective interventions that target frontotemporal function in patients with schizophrenia.

## Introduction

Previous research has demonstrated that individuals with schizophrenia tend to show a lack of insight that affects their symptom, psychosocial functioning, and treatment outcomes (1, 2). However, in 2004, the definition of insight was extended to include the cognitive processes that are involved in patients’ re-evaluation of their anomalous experiences and misinterpretations (3, 4). Beck and colleagues (3, 5) referred to this as cognitive insight, and further identified two underlying components, namely self-reflectiveness (SR) and self-certainty (SC). To examine these factors, the Beck Cognitive Insight Scale (BCIS) was developed (3), which revealed that when the scores for SR are low or when those for SC are high, then an individual’s overall cognitive insight ability is likely impaired.

Since the development of the BCIS, many studies have evaluated the relationship between the BCIS scores and delusions (3, 5, 6), while others have examined the relationship between the BCIS scores and anxiety (7), depression (8), negative symptoms (9, 10), and functional outcome (11–13). However, recently, interest in research on the neural correlates of cognitive insight in patients with schizophrenia has been increasing (14). Studies on this topic suggest that the ventrolateral prefrontal cortex (VLPFC) may be particularly important for cognitive insight in individuals with schizophrenia (14). For instance, higher SR has been linked to increased neural activation in the right ventrolateral PFC of individuals with schizophrenia (15). Moreover, in a previous near-infrared spectroscopy (NIRS) study performed in our laboratory, we found that SR modulated right ventrolateral PFC and right temporal functions during verbal fluency task (VFT) in people with schizophrenia (16). Interestingly, we also revealed that the ventrolateral PFC and other PFC regions played a significant role in the subjective quality of life (QOL) of individuals with schizophrenia (17, 18). These findings suggest that ventrolateral PFC function may mediate the relationship between cognitive insight and subjective QOL in schizophrenia. However, this hypothesis has not been fully examined.

Near-infrared spectroscopy is a comparably new neuroimaging technique that has received increasing attention in the field of neuroscience and psychiatry. NIRS is a non-invasive, high time resolution (0.1 s) functional optical technique revealing the spatiotemporal characteristics of brain functioning by using near-infrared light (19, 20). In contrast to other neuroimaging methodologies such as functional magnetic resonance imaging (fMRI) and electroencephalography (EEG), NIRS can be measured under a more restraint-free environment that is especially suitable for psychiatric patients. This has made it feasible to perform NIRS in real-world clinical settings (21). In NIRS, typical cortical activation represents not only decreased concentration of deoxy-hemoglobin (deoxy-Hb), which is considered the main source of blood oxygenation level-dependent (BOLD) contrast increase in fMRI, but also a relatively larger increase in oxy-hemoglobin concentration (oxy-Hb) (21). NIRS measurement during a VFT was recently approved by the Ministry of Health, Labor, and Welfare of Japan as an advanced medical technology for the aid of differential diagnosis of depressive state psychiatric illnesses and has been frequently applied in clinical settings in Japan (21). In addition, several reports suggest that the mean oxy-Hb changes induced by a VFT in patients with schizophrenia are significantly decreased compared with those observed in controls (22–26).

Cognitive insight has been found to predict positive gains in individuals undergoing psychotherapy for psychosis, and improvements in cognitive insight have been correlated with improvements in delusional beliefs (12, 27). However, better cognitive insight has also been linked to negative outcomes (8, 28, 29). Weintraub and Weisman de Mamani (30) recently suggested that cognitive insight might have a similarly equivocal relationship with subjective QOL in a subclinical sample, although the exact nature of the relationship between cognitive insight and subjective QOL for patients with schizophrenia has yet to be determined.

To examine these issues, we concurrently assessed frontotemporal function, cognitive insight, and subjective QOL in patients with schizophrenia. Our three hypotheses were as follows: (1) relative to healthy controls, patients with schizophrenia would have detectable abnormalities in VFT-related frontotemporal function; (2) cognitive insight would be related to the observed frontotemporal (specifically the right) function and subjective QOL; and (3) right frontotemporal function would mediate the relationship between cognitive insight and subjective QOL.

## Materials and Methods

### Participants

This study was approved by the Ethics Committee of Tottori University Faculty of Medicine (approval No. 885), and the investigation was conducted in accordance with the latest version of the Declaration of Helsinki. Written informed consent was obtained from each participant after the study procedures had been explained.

The participants included 32 patients with schizophrenia who were clinically stable enough to undergo the assessments and 32 age- and gender-matched healthy controls (Table 1). All the patients were receiving second-generation antipsychotic medication during the study, and their chlorpromazine-equivalent daily doses were calculated and are shown in Table 1 (monotherapy/two drugs therapy: 28/4; 10 aripiprazole, 8 blonanserin, 8 olanzapine, 3 risperidone, 3 perospirone, 2 paliperidone, 2 quetiapine). The patients were recruited from the outpatient population of Tottori University Hospital and were diagnosed by the same experienced psychiatrists (Masaaki Iwata and Koichi Kaneko) using the criteria specified in the Diagnostic and Statistical Manual of Mental Disorders, Fourth Edition (31). On the day of the NIRS experiment, patients’ psychiatric symptoms were evaluated by the same psychiatrists (Masaaki Iwata and Koichi Kaneko) using the Positive and Negative Syndrome Scale (32).

**Table 1:** Demographics and clinical characteristics of the participants.

	Patients with schizophrenia (mean ± SD)	Healthy controls (mean ± SD)	Comparison between groups
Sex, *n* (female/male)	32 (24/8)	32 (21/11)	χ^2^ = 0.674, *p* = 0.412
Age, years	31.3 ± 9.8	31.9 ± 10.9	*t*(df = 62) = 0.253, *p* = 0.801
Handedness	96.7 ± 13.0	97.7 ± 10.8	*t*(df = 62) = 0.334, *p* = 0.739
Education, years	13.7 ± 2.2	14.7 ± 2.2	*t*(df = 62) = 1.822, *p* = 0.073
Estimated premorbid IQ	98.6 ± 9.4	100.3 ± 8.3	*t*(df = 62) = 0.744, *p* = 0.460
Duration of illness, years	9.7 ± 6.8	–	–
Chlorpromazine-equivalent dose, mg/day	488.9 ± 315.9	–	–
**PANSS**			
Positive	13.8 ± 4.0	–	–
Negative	18.1 ± 5.3	–	–
General psychopathology	31.6 ± 9.1	–	–
Number of words generated	11.9 ± 3.7	14.3 ± 3.8	*t*(df = 62) = 2.496, *p* = 0.015
**BCIS**			
Self-reflectiveness	11.2 ± 3.7	–	–
Self-certainty	5.3 ± 3.1	–	–
**SF-36**			
Physical functioning	46.8 ± 13.7	–	–
Role limitations—physical	38.1 ± 15.6	–	–
Bodily pain	48.1 ± 12.7	–	–
General health	40.6 ± 12.8	–	–
Vitality	41.6 ± 12.9	–	–
Social functioning	42.9 ± 14.6	–	–
Role limitations—emotional	36.1 ± 15.2	–	–
Mental health	40.7 ± 12.6	–	–

*df, degrees of freedom; IQ, intelligence quotient; PANSS, Positive and Negative Symptom Scale; BCIS, Beck Cognitive Insight Scale; SF-36, Medical Outcomes Study 36-item Short-Form Health Survey*.

All participants were right-handed according to the Edinburgh Handedness Inventory (33). The participants in this study partially overlapped with those in our previous studies (16–18), but were not identical.

### Assessments

#### Cognitive Insight

Cognitive insight was assessed with the BCIS (3, 10), a 15-item self-report inventory. The BCIS consisted of the following two components: SR and SC. The former includes items measuring objectivity, reflectiveness, and openness to feedback, whereas the latter measures the certainty about one’s own beliefs or judgment. The BCIS was administered to only the patients.

#### Subjective QOL Measurement

All patients completed a self-assessment of QOL using the Medical Outcomes Study 36-item Short-Form Health Survey (SF-36) (34, 35). The SF-36 is scored such that 8 scale scores are given: physical functioning, role physical, bodily pain, general health perceptions, vitality, social functioning, role emotional, and mental health. The SF-36 Scoring Manual (36) does not provide support to calculate a single measure of health-related QOL, such as an “SF-36 Total/Global/Overall Score.” Therefore, we only adopted the subscale scores, which were transformed to make a minimum and maximum possible score of between 0 and 100, where higher scores indicate better health and well-being.

#### NIRS Measurements (37)

The 52-channel NIRS machine measures relative changes in oxy-hemoglobin (oxy-Hb) and deoxy-hemoglobin (deoxy-Hb) using two wavelengths (695 and 830 nm) of infrared light based on the modified Beer–Lambert law (37, 38). The arrangement of the source-detector probes enabled us to measure the Hb values from both the PFC and temporal regions(39, 40).

#### Cognitive Task

The task procedure used in this study was similar to that described by Takizawa et al. (24) in that Hb changes were measured during a letter version of the VFT. When performing the task, the participant sat on a comfortable chair and was instructed to minimize movements, such as head movements, jaw clenching, and eye blinking, to avoid producing artifacts during the NIRS measurements. The 160-s block-design VFT was divided into the following three periods: 30-s pre-task period, 60-s task period, and 70-s post-task period. The total number of correct words generated during the VFT was adopted as a measure of task performance (16).

**Figure 1:**
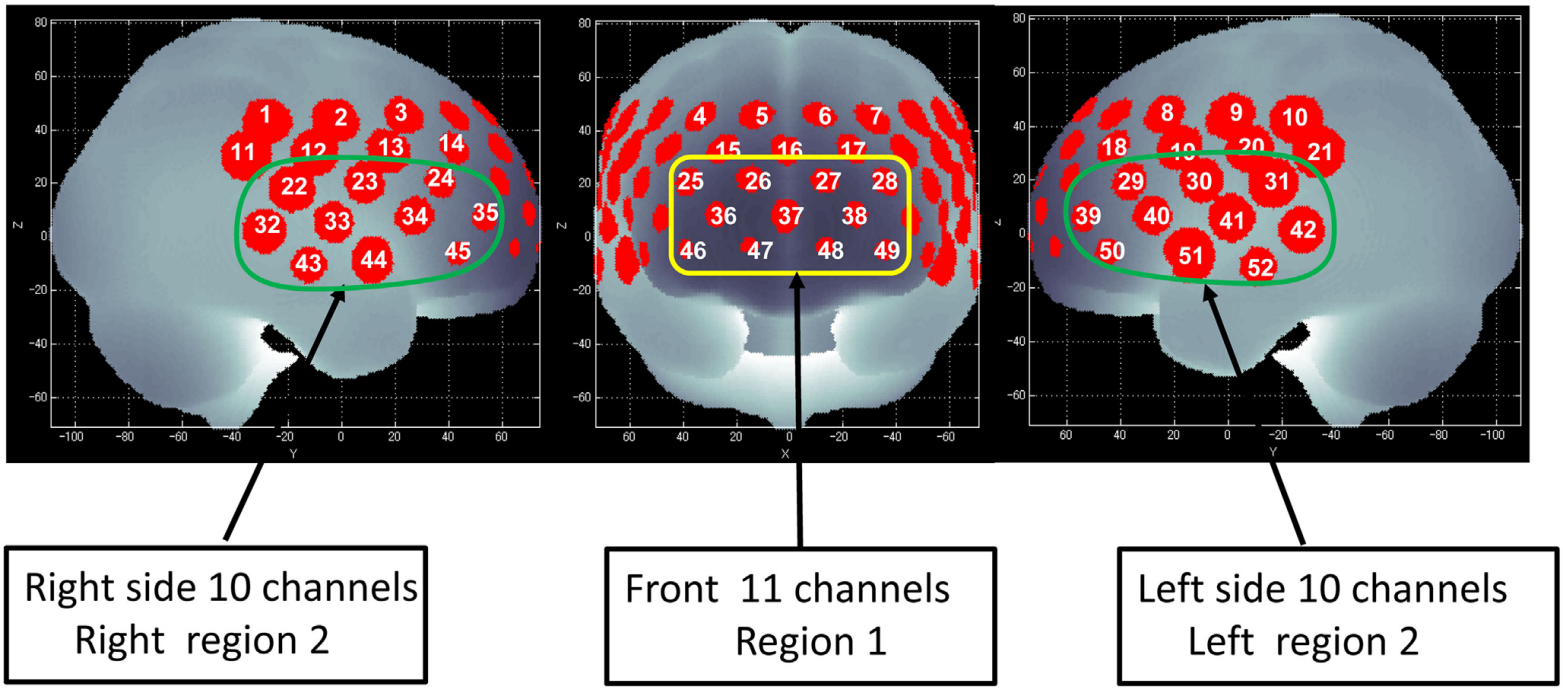
Channel positions on the brain surface. Right region 2 consists of the right 10 channels (22–24, 32–35, and 43–45) and is located approximately on the right ventrolateral prefrontal cortex (VLPFC) and anterior part of the temporal cortex (aTC) region. Left region 2 consists of the left 10 channels (29–31, 39–42, and 50–52) and is located approximately on the left VLPFC and aTC region. Region 1 consists of the center 11 channels (25–28, 36–38, and 46–49) and is located approximately on the dorsolateral prefrontal cortex (DLPFC) and frontopolar cortex (FPC) region (45).

The 160-s block-design VFT contains three different time periods: a 30-s pre-task period, a 60-s task period, and a 70-s post-task period (Figure 1). For the pre- and post-task baseline periods, the subjects were instructed to consecutively repeat the five Japanese vowels (“a,” “i,” “u,” “e,” and “o”) aloud. As readout from NIRS, the contrast between the verbal fluency condition and the vocalization condition was used to increase specificity for the verbal fluency canceling out the vocalization effect. During the task period, they were instructed to generate as many Japanese words beginning with a designated syllable as possible. The three sets of initial syllables (A; /to/, /se/, /o/, B; /a/, /ki/, /ha/, C; /na/, /i/, /ta/) were presented in counterbalanced order among the subjects and each syllable changed every 20 s during the 60-s task.

The sampling frequency was 10 Hz. To examine VFT task-related activation, data were analyzed using the “integral mode” installed on the NIRS machine, in which the pre-task baseline was calculated as the mean over the 10-s period immediately prior to the task period, and the post-task baseline was calculated as the mean over the 5-s period that followed after the 50-s post-task period. Linear fitting was applied to the data recorded between these two baselines. A moving-average method, using a 5-s window, was applied to remove any short-term motion artifacts. In addition, we rejected noise related to body-movement artifacts (no signal, high frequency, and low frequency) using the algorithm published by Takizawa et al. (21).

### Regions of Interest

We defined each measuring area between pairs of source-detector probes as “channel.” It is supposed that the NIRS machine, in which the source-detector spacing is 3.0 cm, measures points at 2–3 cm depth from the scalp, i.e., the surface of the cerebral cortex (41–43). The probes of the NIRS machine were fixed with thermoplastic 3 × 11 shells, with the lowest probes positioned along the Fp1–Fp2 line according to the international 10–20 system used in EEG. The 52 measuring areas are labeled ch1–ch52 from the right-posterior to the left-anterior.

Of the 52 NIRS channels, region 1 included channels 25–28, 36–38, and 46–49. The right side of region 2 included channels 22–24, 32–35, and 43–45, while the left side of region 2 included channels 29–31, 39–42, and 50–52 (Figure 1). The NIRS signal of region 1 consisted of the signals from channels located approximately in the dorsolateral PFC and frontopolar cortex (FPC) [dorsolateral prefrontal cortex (DLPFC)/FPC; i.e., the superior and middle frontal gyrus]. Region 2 consisted of signals from channels located approximately in the ventrolateral PFC and the anterior part of the temporal cortex (VLPFC/aTC) (21, 44, 45).

### Statistical Analysis

Statistical analyses were performed using SPSS Statistics 24.0 (IBM Corporation, Armonk, NY, USA). Hemodynamic responses during the VFT in region 1 and left and right region 2 were assessed by the “integral value” of Hb changes. We used the integral value of the oxy-Hb (as opposed to deoxy-Hb) changes that occurred during the VFT, as an index of cortical activity, because oxy-Hb better reflects this activity and is better correlated with fMRI BOLD signals (46–48) compared with deoxy-Hb.

First, the integral value of the oxy-Hb changes that occurred during the task period were compared between the participant groups using Student’s *t*-tests [with a Bonferroni-corrected alpha level of 0.0167 (0.05/3)]. When there was a significant between-group difference in the performance level, we performed additional analyses of covariance using the performance level as a covariate to the integral value of oxy-Hb changes. Next, Pearson’s product–moment correlation coefficients adopting the false discovery rate (FDR) method were calculated to test the correlations between the integral value of oxy-Hb changes in each region of interest (ROI; region 1 and left and right region 2) and the BCIS subscale scores (SR and SC) and SF-36 subscale scores.

A mediation analysis was performed using Hayes’ PROCESS macro, a regression-based path analysis technique (49). Using an ordinary least-squares framework, PROCESS estimates the direct and indirect effects in mediator models. To test the mediation hypotheses, PROCESS uses bootstrapping to construct confidence intervals (CIs) for indirect effects through repeated sampling of the data set. Findings are based on 5,000 bias-corrected bootstrapped samples. In the event that 0 does not lie within the 95% CI for the bootstrapped results for indirect effects, we can conclude that the indirect effect is significantly different from 0 and that mediation is demonstrated (50).

## Results

The participants’ demographic data are shown in Table 1. Patients with schizophrenia had a significantly lower performance level on the VFT (number of words generated) compared with healthy controls (*t* = 2.496, *p* = 0.015).

### Frontotemporal Activation

**Figure 2:**
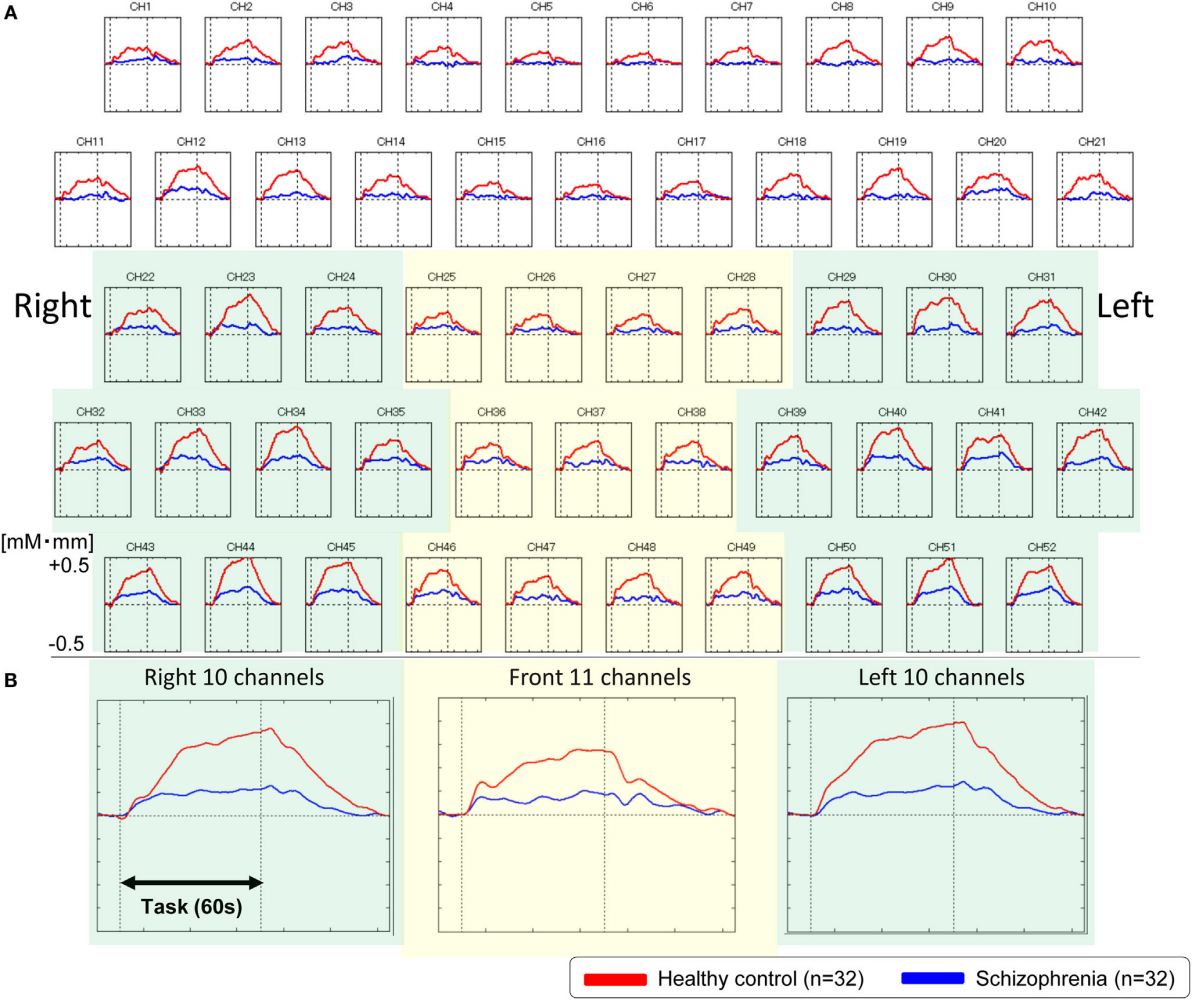
Waveforms of oxygenated-hemoglobin (oxy-Hb) concentration changes during the VFT. **(A)** The upper figures show the overall average waveforms of concentrations of oxy-Hb changes during the VFT in patients with schizophrenia (blue) and healthy controls (red). **(B)** In the lower three figures, the right figure shows the average waveforms of oxy-Hb changes for the 10 channels in the right VLPFC/aTC region, the center figure shows the average waveforms of oxy-Hb changes for the 11 channels in the frontal pole region, and the left figure shows the average waveforms of oxy-Hb changes for the 10 channels in the left VLPFC/aTC region. Abbreviations: VLPFC, ventrolateral prefrontal cortex; aTC, anterior part of the temporal cortex; VFT, verbal fluency task.

We used NIRS to evaluate our first hypothesis that patients with schizophrenia would have abnormalities in frontotemporal regions. The overall mean oxy-Hb change waveforms for the 52 channels and three ROIs in each group are shown in Figure 2. Compared with healthy controls, patients with schizophrenia exhibited significantly smaller integral values of oxy-Hb changes (region 1: *t* = 4.177, *p* < 0.001; left region: *t* = 4.923, *p* < 0.001; right region 2: *t* = 4.027, *p* < 0.001). The between-group differences in the integral values of oxy-Hb changes remained significant after correcting for the performance level in the three ROIs (region 1: *F* = 14.907, *p* < 0.001; left region: *F* = 21.261, *p* < 0.001; right region 2: *F* = 14.327, *p* < 0.001), according to analyses of covariance using performance on the VFT as a covariate to the integral values of oxy-Hb changes.

### Correlation Analyses

Next, we examined whether cognitive insight was related to frontotemporal function and subjective QOL in patients with schizophrenia. To test this hypothesis, we extracted the integral values of oxy-Hb changes from the three ROIs (region 1 and left and right region 2), where patients with schizophrenia showed reduced VFT-related neural activity compared with healthy controls, and conducted Pearson’s correlations with the cognitive insight and subjective QOL scores (Table 2).

**Table 2:** Correlations between the VFT-related hemodynamic responses and measures of cognitive insight and subjective QOL in patients with schizophrenia.

	Cognitive insight		Region 1 (DLPFC/FPC)	Left region 2 (left VLPFC/aTC)	Right region 2 (right VLPFC/aTC)
	Self-reflectiveness	Self-certainty			
**Frontotemporal region**					
Region 1 (DLPFC/FPC)	0.337	−0.100	–	–	–
Left region 2 (left VLPFC/aTC)	0.273	0.011	–	–	–
Right region 2 (right VLPFC/aTC)	0.506***	−0.163	–	–	–
**Subjective QOL**					
Physical functioning	−0.171	0.079	0.128	0.135	0.060
Role limitations—physical	0.043	−0.075	0.050	0.072	0.188
Bodily pain	0.020	−0.312	0.117	0.239	0.232
General health	0.121	−0.241	0.287	0.392*	0.378
Vitality	0.411*	−0.057	0.495***	0.492*	0.501***
Social functioning	0.226	−0.082	0.301	0.222	0.120
Role limitations—emotional	0.183	−0.094	0.213	0.168	0.080
Mental health	0.508***	−0.047	0.320	0.461**	0.509***

*VFT, verbal fluency task; QOL, quality of life; DLPFC/FPC, dorsolateral prefrontal cortex and frontopolar cortex; VLPFC/aTC, ventrolateral prefrontal cortex and the anterior part of the temporal cortex*.

*p < 0.05.

**p < 0.01.

***p < 0.005.

In patients with schizophrenia, better cognitive insight, according to the SR subscale of the BCIS was related to a higher subjective QOL (vitality: *r* = 0.411, FDR-corrected *p* = 0.013; mental health: *r* = 0.508, FDR-corrected *p* = 0.006) and to increased right region 2 VFT-related activity (right VLPFC/aTC; *r* = 0.506, FDR-corrected *p* = 0.017). A higher subjective QOL was also related to increased VFT-related activity in region 1 (DLPFC/FPC; vitality: *r* = 0.495, FDR-corrected *p* = 0.006), left region 2 (left VLPFC/aTC; vitality: *r* = 0.492, FDR-corrected *p* = 0.006; mental health: *r* = 0.461, FDR-corrected *p* = 0.013), and right region 2 (right VLPFC/aTC; vitality: *r* = 0.501, FDR-corrected *p* = 0.013; mental health: *r* = 0.509, FDR-corrected *p* = 0.006), but neither region 1 (*r* = 0.273, FDR-corrected *p* > 0.50) nor left region 2 (*r* = 0.337, FDR-corrected *p* > 0.05) were related to SR.

However, there were no significant correlations between the SC and other subscale of QOL or all ROIs VFT-related activity (FDR-corrected *p* > 0.05).

### Mediation Analyses

**Figure 3:**
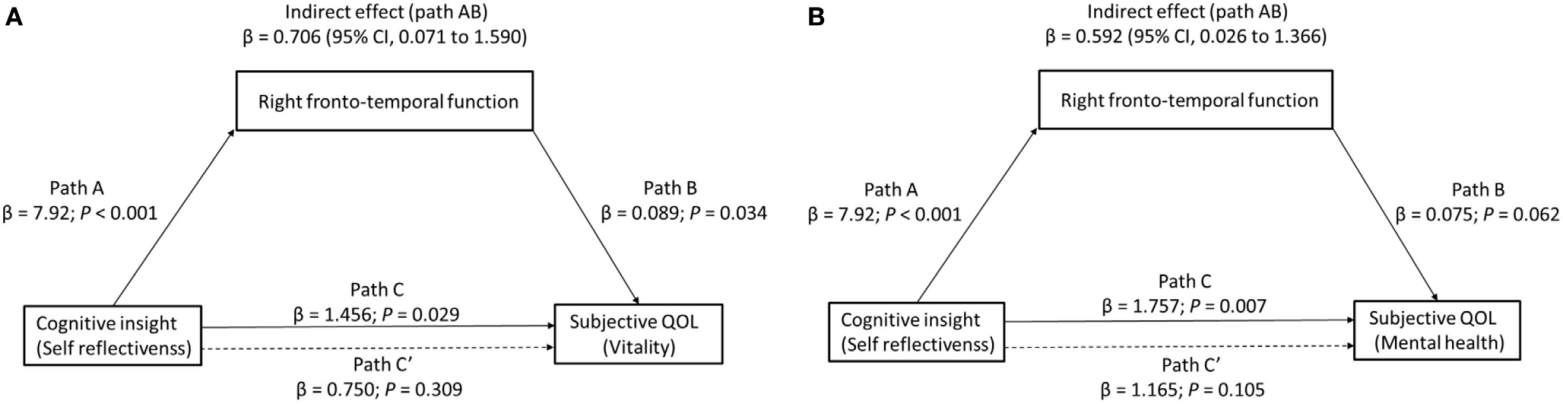
The effect of cognitive insight on subjective quality of life (QOL) through right frontotemporal function. Path C represents the variance in the level of cognitive insight that is associated with the subjective QOL (**(A)** vitality or **(B)** mental health) level, and Path C′ represents the association between the level of cognitive insight and subjective QOL after taking into account right frontotemporal function as a mediator. When right frontotemporal function was included in the model, the direct effect of cognitive insight (self-reflectiveness) on subjective QOL (dashed line) (left: vitality, right: mental health) was no longer significant, indicating a fully mediated effect. Path AB is the mediation effect and is significant at *p* < 0.05 based on confidence intervals from bias-corrected bootstrapping of 5,000 samples.

Finally, we determined whether right frontotemporal function mediated the relationship between cognitive insight and subjective QOL. Because the vitality and mental health subscales were found to relate to both right region 2 (right VLPFC/aTC) VFT-related activity and our measure of cognitive insight (SR), to test our third hypothesis that right VLPFC/aTC function mediates the relationship between cognitive insight and subjective QOL, we entered right VLPFC/aTC VFT-related activity, SR, and vitality or mental health scores into a single mediator model. For the mediation analysis to be justified, the predictor, mediator, and outcome variables must all be inter-related (51, 52). Indeed, all four paths were in the predicted direction (Figure 3). Cognitive insight had a positive effect on subjective QOL (vitality: β = 1.456, *p* = 0.029; mental health: β = 1.757, *p* = 0.007) and right region 2 VFT-related activity (β = 7.920, *p* < 0.001), and right region 2 VFT-related activity had a positive effect on subjective QOL (vitality: β = 0.089, *p* = 0.034; mental health: β = 0.075, *p* = 0.062). Bootstrap analysis of the indirect effect revealed bias-corrected CIs excluding 0 (vitality: β = 0.71, SE = 0.39, 95% CI = 0.071–1.59; mental health: β = 0.59, SE = 0.33, 95% CI = 0.03–1.37). Importantly, the direct effect of cognitive insight on subjective QOL, after controlling for right VLPFC/aTC function, was no longer significant (vitality: β = 0.75, 95% CI = −0.730 to 2.229; mental health: β = 1.17, 95% CI = −0.259 to 2.589), indicating that right VLPFC/aTC function fully mediated the relationship between cognitive insight in the SR domain and subjective QOL.

## Discussion

This study had three main findings. First, we identified regions within our selected frontotemporal area (all ROIs) where participants with schizophrenia had reduced hemodynamic responses compared with healthy controls. This finding is consistent with those of prior studies (16, 22–26) and provides further evidence that patients with schizophrenia exhibit abnormalities in frontotemporal regions. Second, the level of cognitive insight as well as the level of subjective QOL were related to neural activity in the right VLPFC/aTC regions, demonstrating a relationship between the neurobiological characteristics of schizophrenia and cognitive insight and subjective QOL. Third, VFT-related neural activity in the right VLPFC/aTC regions fully mediated the relationship between cognitive insight and subjective QOL, indicating that the disease-related level of cognitive insight may affect the subjective QOL through abnormalities in frontotemporal function.

Collectively, our findings have implications for understanding the specific role the cognitive insight level (particularly SR) plays in the level of subjective QOL in individuals with schizophrenia. Recent functional (fMRI and NIRS) (16, 53) and structural MRI (54) studies highlighted the relationship between SR and the right ventrolateral PFC. SR is defined as the ability to simultaneously consider various types of information, perspectives, and alternative hypotheses to generate judgments about the self, and this ability utilizes verbal working memory and decision-making processes (16, 54). High levels of SR may encourage patients to doubt their distorted and unrealistic perceptions or thoughts, leading them to have a more-objective attitude toward their illness (55). For instance, Phalen et al. (2) (p. 840) stated that “while engaged in treatment, those patients with higher cognitive insight may be better able to incorporate the feedback of mental health professionals and consider alternative ways of thinking” (56). Trials employing cognitive behavior therapy for psychosis support this theory, as they have consistently found that better cognitive insight is predictive of better responses to psychosocial treatments (2, 27). Moreover, in decision-making, the right ventrolateral PFC plays a role generating alternative perspectives in tasks requiring individuals to respond to a problem that has various potential answers (57, 58). In the context of SR, one’s willingness to admit fallibility and corrigibility and to recognize dysfunctional reasoning may in part depend on the controlled retrieval of information from memory, which is mediated by the ventrolateral PFC (15, 53). In our prior NIRS studies, we identified a relationship between the ventrolateral PFC and other PFC areas and subjective QOL in patients with schizophrenia (17, 18). Here, we found that the right VLPFC/aTC fully mediated the relationship between SR and subjective QOL, implying that this region is critical for both functions. This findings confirm our prior findings, while the results from the mediation analysis additionally demonstrate that right ventrolateral PFC function is one of the mechanisms underlying the relationship between cognitive insight (particularly SR) and subjective QOL.

Although cognitive insight is commonly considered an important factor in schizophrenia (2, 59), how cognitive insight relates to broader outcomes like QOL remains unknown. A well-replicated pattern in schizophrenia research is that higher levels of cognitive insight are often associated with better outcomes [e.g., Ref. (30, 60)]. Our findings imply that higher SR levels may be generally associated with better subjective QOL. However, this association is not necessarily consistent. Some lines of evidence suggest that superior cognitive insight is related to positive outcomes for patents with psychotic disorders (8, 12, 27), while others imply that it is associated with negative outcomes (8, 28, 29). One possible explanation for these equivocal findings is that the effect of cognitive insight on QOL depends on the presence of other variables. Recently, Phalen et al. (2) found that SR had an unmoderated positive relationship with QOL and that the effect of SC on QOL was moderated by symptom severity. The authors suggested that cognitive insight is related to QOL, but that different aspects of cognitive insight may relate to QOL in different ways (2). According to their view, it is likely that symptom severity moderates the effects of cognitive insight on QOL because the flexible perspective shifting abilities associated with better cognitive insight may differ in patients with varying levels of symptom severity (2). For patients whose symptoms are severe and obvious to others, higher SC may serve as a protective factor against the social stigma that may harm the QOL (2). On the other hand, SR may generally be associated with better QOL as noted above. However, the meta-analysis by Palmer et al. (8) supports the alternative view of Kim et al. (4) that SR is negatively associated with the level of subjective QOL. Thus, further studies are needed to clarify the relationship between SR and QOL. Overall, our findings suggest that cognitive insight (SR) is related to subjective QOL; it is possible that SR contributes to improved subjective QOL (vitality, mental health) *via* the neural activity in the right VLPFC/aTC. Additional studies are necessary to explore other possible mediating and moderating factors and to evaluate the effects that various therapeutic interventions may have on the relationship between cognitive insight and QOL.

The two subscales that showed a positive association with oxy-Hb changes, vitality and mental health, were closely related to mental aspects of QOL, which was similar to the motivation/energy subscale showing a positive relationship with left frontal and temporal gray matter volume in Ubukata et al. (61). Interestingly, executive functioning, a cognitive process involved in VFT, has been reported to show a positive relationship with different aspects of subjective QOL from those found in this study, which are self-evaluation of side-effects and symptoms (62, 63). These findings suggest a possibility that oxy-Hb changes elicited by VFT may reflect the motivation and positive engagement of the task rather than cognitive ability *per se* (18).

Our findings need to be interpreted within the context of the study limitations. First, multichannel NIRS has limited spatial resolution compared with fMRI (~1 mm). However, a recent MRI and NIRS combination study, which used a method for the probabilistic registration of NIRS data onto the Montreal Neurological Institute coordinate space, suggested that the errors of spatial estimation, expressed as SDs, were approximately 10 mm (40, 64). Second, the relationship among cognitive insight, QOL in the mental aspects and right frontotemporal activities was observed only in patients with schizophrenia, and therefore care must be taken that it cannot be applied generally to other populations.

In conclusion, this study demonstrated that the level of cognitive insight (especially SR) affected the subjective QOL level in patients with schizophrenia owing to abnormalities in VFT-related frontotemporal function. These findings improve our understanding of how the cognitive insight indicators of schizophrenia are related to the clinical and behavioral presentations of the illness.

## Ethics Statement

This study was approved by the ethics committee of the Faculty of Medicine of Tottori University (approval No. 885) and the investigation was conducted in accordance with the latest version of the Declaration of Helsinki. Written informed consent was obtained from each participant after the study procedures had been explained.

## Author Contributions

SP, KN, and KK designed the study; SP acquired and analyzed the data; SP and KN wrote the first draft of the article; SP, KN, MSI, HO, MKI, IN, and KK contributed to the interpretation of the results and the writing of the manuscript. All authors have approved the final manuscript.

## Conflict of Interest Statement

The authors declare that the research was conducted in the absence of any commercial or financial relationships that could be construed as a potential conflict of interest.
